# Image Reconstruction with Maclaurin Series Expansion

**DOI:** 10.33425/2769-6294.1041

**Published:** 2025-08-10

**Authors:** Gengsheng L Zeng

**Affiliations:** Department of Computer Science, Utah Valley University, Orem, USA.

**Keywords:** Functions with finite support, Entire function, Inverse problem, Taylor series expansion, Fourier transform, Image reconstruction, Tomography, Mix high-order partial derivatives, Central slice theorem, Approximation, Data sufficiency conditions

## Abstract

This is a forward-thinking theoretical investigation and may not have practical values for current imaging systems. This investigation assumes that there is no noise in the measurements, the signals are continuous (not sampled), the computer has perfect precession, and there are no round-off errors. Under these unrealistic conditions, we can form a Maclaurin series expansion in the Fourier domain with measurements in a small scanning angular range. We show that this Maclaurin series expansion converges in the entire Fourier space. As a result, a complete data set is available for image reconstruction. The Fourier domain is complex; the expansion coefficients are most likely complex with real parts and imaginary parts. Computer simulations are performed to illustrate a 2D spatial-domain image can be obtained if a Fourier-domain truncated Maclaurin series expansion is available. Our goal is to use minimum data for trust-worthy reconstruction without any prior knowledge and training data.

## Introduction

There are many data sufficiency conditions for various imaging modalities and imaging geometries [1–15]. For example, in two-dimensional (2D) imaging, the parallel-beam system requires a scanning angular range of 180°. The fan-beam system requires a scanning angular range of 180° plus the fan angle. If the scanning trajectories satisfy the data sufficiency conditions, we have stable image reconstruction algorithms, which can be analytical or iterative.

Even when the angular range satisfied the data sufficiency conditions, the sampling on the detector may not be adequate. If the detector is not large enough to cover the object to be imaged, the projection data is truncated, resulting in an under-sampling situation [16–33]. Another under-sampling situation is that the angular sampling is not dense enough, which is also known as few-view tomography [33–49].

When data is insufficient, some other assumes can make the inverse problem solvable. One of such situations is compressed sensing [50–60]. The compressed sensing methods consider the inverse problem solutions, which are sparse, that is, most of the elements are zero. In tomographic application, the solutions x can be assumed as piecewise constant. The derivative of x of the finite difference of x is a sparse image. The compressed sensing theory suggests that a usable sparse solution can be obtained by minimization of the ${\text{L}}_{0}$ norm of the sparse solution x. For a piecewise-constant solution, we minimize the ${\text{L}}_{0}$ norm of the finite difference of x. The ${\text{L}}_{0}$ norm minimization is not an easy task, because the ${\text{L}}_{0}$ norm of an image is the total count of non-zero image pixels. The gradient of this total count with respect to each pixel does not exist. The gradient-based optimization algorithms do not work.

A practical work around is to use the ${\text{L}}_{1}$ norm to approximate the ${\text{L}}_{0}$ norm. For a piecewise-constant image, this remedy leads to the total variation (TV) minimization algorithms. In this paper, we do not assume that the images are piecewise constant. We do not treat the image reconstruction problem as a compressed sensing problem. We do not discretize the imaging problem. We assume the image and its projection measurements are continuous.

At the beginning of this section, we state that a data sufficiency condition for a 2D parallel-beam imaging problem is to acquire data over an angular range of 180°. We argue that this data sufficiency condition is not necessary. This condition is derived based on the assumption that the entire Fourier space must be completely measured. We will show in the next section that we do not have to measure the entire Fourier space to capture the complete information about the spatial-domain object. In fact, we only need to know the Fourier transform at one location, for example, at the center.

This paper assumes a perfect ideal world, where the detected signals are continuous and noiseless. As will be shown in the next section, perfect high-order mixed partial derivatives can be calculated by the measurements. All these assumptions guarantee that we can form a 2D Taylor series expansion in the Fourier domain of the object. This paper is forward-thinking, and we may not be able to implement the proposed system using today’s technology. It is likely that we are unable to do computer simulations for the proposed system due to the round-off errors and finite word-length limits in a practical computer. The errors may cause the Taylor series expansion unstable or even diverge.

For line-integral based imaging systems such as x-ray computer tomography (CT), positron emission tomography (PET), and single photon emission computed tomography (SPECT), it is challenging to estimate the derivatives in the Fourier domain. We will use the Central Slice Theorem to suggest some potential strategies in the next section.

## Methods

In mathematics, a “compact support” refers to a function that is only non-zero within a bounded, closed set (a compact set), meaning it becomes zero outside of that specific region. In medical imaging, the patient’s body always has compact support.

If a function has a compact support, the Fourier transform of this function is an “entire function” (also known as holomorphic function and analytic function). An entire function has many desirable properties. An entire function can be differentiated with any order at any point in the complex plane and has no singularities. The Taylor series expansion of an entire function converges everywhere in the complex plane.

Let 0≤f(x,y)≤M be a 2D real function that has finite support. Assume that f(x,y) vanishes for |x|≥R and |y|≥R According to the Paley-Wiener theorem [61], for a square-integrable function with a finite support, its Fourier transform is holomorphic.

The 2D Fourier transform of f(x,y) is given as

(1) $$F\left(u,v\right)=\overset{R}{\underset{-R}{\int }}\overset{R}{\underset{-R}{\int }}f\left(x,y\right)e^{-2\pi j\left(xu+yv\right)}\text{dxdy}\;=\overset{R}{\underset{-R}{\int }}\overset{R}{\underset{-R}{\int }}f\left(x,y\right)\overset{\infty }{\underset{k=0}{\sum }}\frac{{\left[-2\pi j\left(xu+yv\right)\right]}^{k}}{k!}\text{dxdy}\;=\overset{\infty }{\underset{k=0}{\sum }}\frac{{\left(-2\pi j\right)}^{k}}{k!}\int_{-R}^{R}\int_{-R}^{R}{\left(xu+yv\right)}^{k}f\left(x,y\right)\text{dxdy}.$$

Interchanging of integration and summation is allowed because f is supported on a finite region and because the series for the exponential function converges absolutely and uniformly [61].

From (1), we have

(2) $$F\left(0,0\right)=\int_{-R}^{R}\int_{-R}^{R}f\left(x,y\right)\text{dxdy}.$$

We also have

(3) $$\frac{\partial^{n+m}F(0,0)}{\partial u^{n}\partial v^{m}}=\sum_{k=0}^{\infty }\sum_{\begin{array}{c} n+m=k\\ n\geq 0,m\geq 0 \end{array}}\frac{(-2\pi j{)}^{k}}{(k-n-m)!}\int_{-R}^{R}\int_{-R}^{R}x^{n}y^{m}f\left(x,y\right)\text{dxdy}.$$

(4) $$F(u,v)=\sum_{k=0}^{\infty }\sum_{\begin{array}{c} n+m=k\\ n\geq 0,m\geq 0 \end{array}}\frac{u^{n}v^{m}}{n!m!}\frac{\partial^{n+m}F(0,0)}{\partial u^{n}\partial v^{m}}.$$

An alternative expression for this Maclaurin series expansion is

(5) $$F(u,v)=\sum_{k=0}^{\infty }\frac{1}{k!}{\left(u\frac{\partial }{\partial u}+v\frac{\partial }{\partial v}\right)}^{k}F(0,0).$$

From the right-hand side of (3), we learn that if a mixed partial derivative is evaluated in the image domain, the whole image f(x,y) must be used in the calculation. Unfortunately, we do not know of any imaging system that can measure or calculate the double integral in (3).

Our innovative strategy is to use the directional derivatives to estimate the mixed partial derivatives. Let $f_{\theta }(s,t)$ be a rotated version of f(x,y) with a rotation angle θ. The Radon transform (i.e., the parallel-beam projections) is given by

(6) $$p_{\theta }(s)=\int_{-\infty }^{\infty }f_{\theta }(s,t)dt.$$

**Figure 1:**
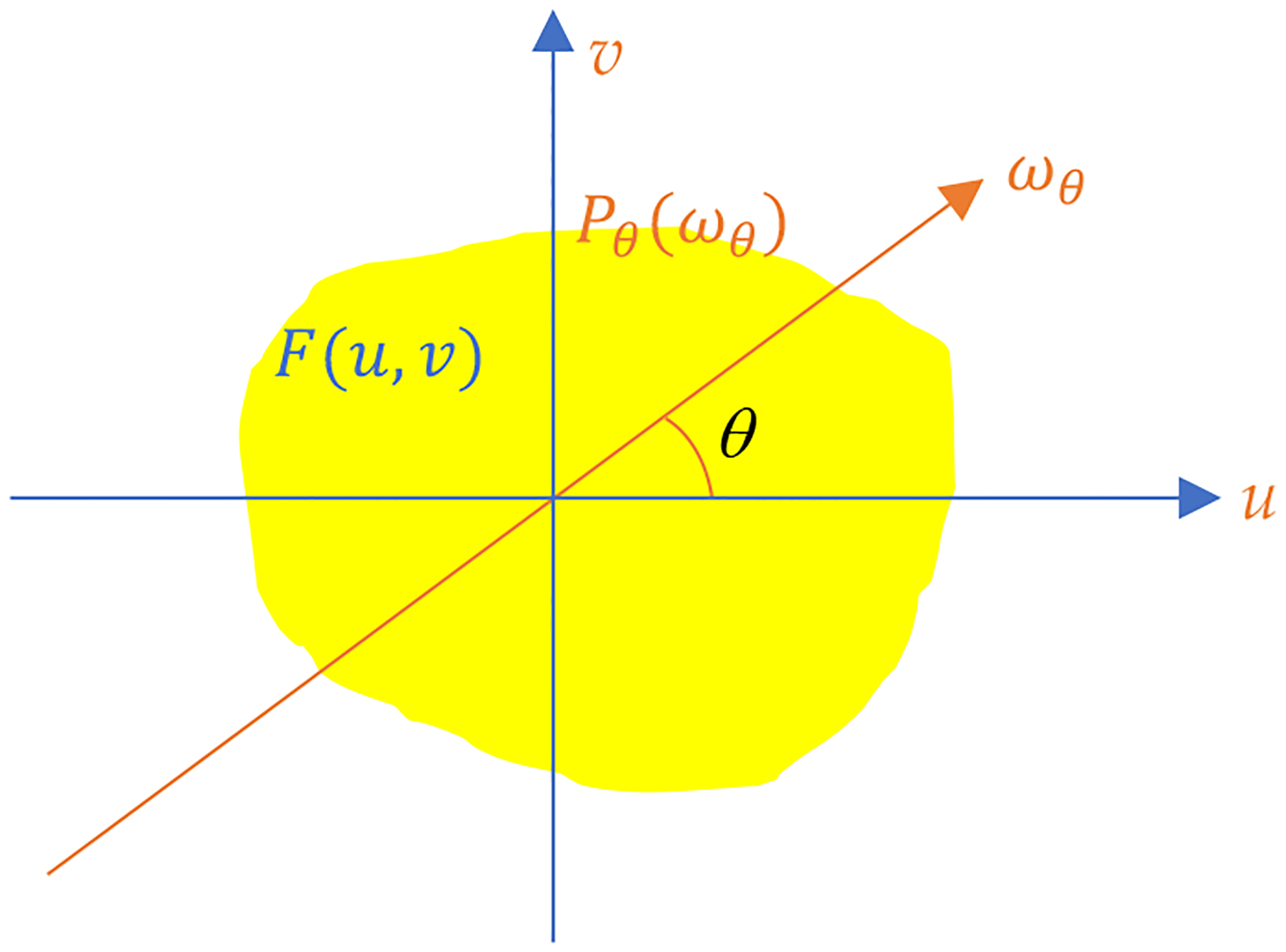
The central slice theorem is the relationship between F(u,v) and $P_{\theta }\left(\omega_{\theta }\right)$, which is a central slice of F(u,v).

The Fourier transform of $p_{\theta }(s)$ with respect to variable s is

(7) $$P_{\theta }\left(\omega_{\theta }\right)=\int_{-\infty }^{\infty }p_{\theta }(s)e^{-2\pi is\omega_{\theta }}ds,$$

where $\omega_{\theta }$ is the frequency variable corresponding to s. Let the 2D Fourier transform of f(x,y) be F(u,v). According to the Central Slice Theorem [1], $P_{\theta }\left(\omega_{\theta }\right)$ is a central slice of F(u,v) as illustrated in Figure 1.

The nth-order directional derivative of $P_{\theta }\left(\omega_{\theta }\right)$ can be calculated as the Fourier transform of the nth moment

(8) $$\frac{d^{n}P_{\theta }\left(\omega_{\theta }\right)}{d\omega_{\theta }^{n}}=\int_{-\infty }^{\infty }s^{n}p_{\theta }(s)e^{-2\pi is\omega_{\theta }}ds.$$

It can be shown that the (n+m)th-order directional derivative of $P_{\theta }\left(\omega_{\theta }\right)$ and the (n+m)th-order mixed partial derivatives $\frac{\partial^{n+m}F(u,v)}{\partial u^{n}\partial v^{m}}$ are related as

(9) $$\frac{d^{k}P_{\theta }\left(\omega_{\theta }\right)}{d\omega_{\theta }^{k}}={\left(cos\theta \frac{\partial }{\partial u}+sin\theta \frac{\partial }{\partial v}\right)}^{k}F\left(u,v\right).$$

We can prove (9) using the mathematical induction method. The version of (9) for k=1 is

(10) $$\frac{dP_{\theta }\left(\omega_{\theta }\right)}{d\omega_{\theta }}=cos\theta \frac{\partial F(u,v)}{\partial u}+sin\theta \frac{\partial F(u,v)}{\partial v},$$

which can be immediately obtained by using the definition of the directional derivative when the partial derivatives $\frac{\partial F(u,v)}{\partial u}$ and $\frac{\partial F(u,v)}{\partial v}$ exist.

We now assume that (9) is valid for an integer k. Then

(11) $$\frac{d^{k+1}P_{\theta }\left(\omega_{\theta }\right)}{d\omega_{\theta }^{k+1}}=\frac{d}{d\omega_{\theta }}\frac{d^{k}P_{\theta }\left(\omega_{\theta }\right)}{d\omega_{\theta }^{k}}\;=\frac{d}{d\omega_{\theta }}\left[{\left(cos\theta \frac{\partial }{\partial u}+sin\theta \frac{\partial }{\partial v}\right)}^{k}F\left(u,v\right)\right]\;\frac{d}{d\omega_{\theta }}\left[\overset{k}{\underset{n=0}{\sum }}\left(\begin{array}{c} n\\ k \end{array}\right)\left(cos^{n}\theta \right)\left({\text{sin}}^{k-n}\theta \right)\frac{\partial^{k}F\left(0,0\right)}{\partial u^{n}\partial v^{k-n}}\right]\;=\overset{k}{\underset{n=0}{\sum }}\left(\begin{array}{c} n\\ k \end{array}\right)\left(cos^{n}\theta \right)\left(sin^{k-n}\theta \right)\frac{d}{d\omega_{\theta }}\frac{\partial^{k}F\left(0,0\right)}{\partial u^{n}\partial v^{k-n}}\;\overset{k}{\underset{n=0}{\sum }}\left(\begin{array}{c} n\\ k \end{array}\right)\left(cos^{n}\theta \right)\left(sin^{k-n}\theta \right)\left(cos\theta \right)\frac{\partial^{k+1}F\left(0,0\right)}{\partial u^{n+1}\partial v^{k-n}}+\overset{k}{\underset{n=0}{\sum }}\left(\begin{array}{c} n\\ k \end{array}\right)\left(cos^{n}\theta \right)\left(sin^{k-n}\theta \right)\left(sin\theta \right)\frac{\partial^{k+1}F\left(0,0\right)}{\partial u^{n}\partial v^{k+1-n}}\;=\left(cos^{k+1}\theta \right)\frac{\partial^{k+1}F\left(0,0\right)}{\partial u^{k+1}}\;+\overset{k-1}{\underset{n=0}{\sum }}\left(\begin{array}{c} n\\ k \end{array}\right)\left(cos^{n+1}\theta \right)\left(sin^{k-n}\theta \right)\frac{\partial^{k+1}F\left(0,0\right)}{\partial u^{n+1}\partial v^{k-n}}\;+\overset{k}{\underset{n=1}{\sum }}\left(\begin{array}{c} n\\ k \end{array}\right)\left(cos^{n}\theta \right)\left(sin^{k+1-n}\theta \right)\frac{\partial^{k+1}F\left(0,0\right)}{\partial u^{n}\partial v^{k+1-n}}\;+\left(sin^{k+1}\theta \right)\frac{\partial^{k+1}F\left(0,0\right)}{\partial v^{k+1}}\;=\left(cos^{k+1}\theta \right)\frac{\partial^{k+1}F\left(0,0\right)}{\partial u^{k+1}}\;+\overset{k}{\underset{m=1}{\sum }}\left(\begin{array}{c} m-1\\ k \end{array}\right)\left(cos^{m}\theta \right)\left(sin^{k+1-m}\theta \right)\frac{\partial^{k+1}F\left(0,0\right)}{\partial u^{m}\partial v^{k+1-m}}\;\left(\text{Let}\;m=n+1\;\text{above}\right)\;+\overset{k}{\underset{n=1}{\sum }}\left(\begin{array}{c} n\\ k \end{array}\right)\left(cos^{n}\theta \right)\left(sin^{k+1-n}\theta \right)\frac{\partial^{k+1}F\left(0,0\right)}{\partial u^{n}\partial v^{k+1-n}}\;+\left(sin^{k+1}\theta \right)\frac{\partial^{k+1}F\left(0,0\right)}{\partial v^{k+1}}\;=\left(cos^{k+1}\theta \right)\frac{\partial^{k+1}F\left(0,0\right)}{\partial u^{k+1}}\;+\overset{k}{\underset{m=1}{\sum }}\left(\begin{array}{c} m-1\\ k \end{array}\right)\left({\text{cos}}^{m}\theta \right)\left(sin^{k+1-m}\theta \right)\frac{\partial^{k+1}F\left(0,0\right)}{\partial u^{m}\partial v^{k+1-m}}\;+\overset{k}{\underset{m=1}{\sum }}\left(\begin{array}{c} m\\ k \end{array}\right)\left({\text{cos}}^{m}\theta \right)\left(sin^{k+1-m}\theta \right)\frac{\partial^{k+1}F\left(0,0\right)}{\partial u^{m}\partial v^{k+1-m}}\;+\left(sin^{k+1}\theta \right)\frac{\partial^{k+1}F\left(0,0\right)}{\partial v^{k+1}}\;=\left(cos^{k+1}\theta \right)\frac{\partial^{k+1}F\left(0,0\right)}{\partial u^{k+1}}\;+\overset{k}{\underset{m=1}{\sum }}\left(\begin{array}{c} m\\ k+1 \end{array}\right)\left(cos^{m}\theta \right)\left(sin^{k+1-m}\theta \right)\frac{\partial^{k+1}F\left(0,0\right)}{\partial u^{m}\partial v^{k+1-m}}\;+\left(sin^{k+1}\theta \right)\frac{\partial^{k+1}F\left(0,0\right)}{\partial v^{k+1}}\;={\left(cos\theta \frac{\partial }{\partial u}+sin\theta \frac{\partial }{\partial v}\right)}^{k+1}F\left(0,0\right).$$

In the derivation above, we used

(12) mk+1=m-1k+mk,

which can be readily verified by definition.

To recap, Eq. (9) is the relationship between the k th-order directional derivative and the kth-order mixed partial derivatives.

Our goal is to form a 2D truncated Taylor series expansion. This expansion requires mixed partial derivatives to construct its coefficients.

A kth-order directional derivative of the Fourier transform of Radon transform $p_{\theta }(s)$ can be calculated by the Fourier transform of the kth moment by Eq. (8).

There are k+1 mixed partial derivatives of order k. Therefore, we need to measure $p_{\theta }(s)$ at k+1 different angles: $\theta_{1},\theta_{2},\ldots \theta_{k+1}$, and solve a system of linear equations:

(13) P=MD,

where

(14) $$D=\left[\begin{array}{c} \frac{\partial^{k}F(0,0)}{\partial u^{0}\partial v^{k-0}}\\ \frac{\partial^{k}F(0,0)}{\partial u^{1}\partial v^{k-1}}\\ \vdots \\ \frac{\partial^{k}F(0,0)}{\partial u^{k}\partial v^{0}} \end{array}\right],$$

(15) $$P=\left[\begin{array}{c} \frac{d^{k}P_{\theta_{1}}(0)}{d\omega_{\theta_{1}}^{k}}\\ \frac{d^{k}P_{\theta_{2}}(0)}{d\omega_{\theta_{1}}^{k}}\\ \vdots \\ \frac{d^{k}P_{\theta_{k+1}}(0)}{d\omega_{\theta_{k+1}}^{k}} \end{array}\right],$$

(16) $$M=\left[\begin{array}{c} r\left(\theta_{1}\right)\\ r\left(\theta_{2}\right)\\ \vdots \\ r\left(\theta_{k+1}\right) \end{array}\right],$$

(17) rθm=⋯,nkcosnθmsink-nθm,⋯

with m=1,2,⋯,k+1 and n=0,1,⋯,k.

The Taylor coefficients are then determined by the measurements at these k+1 angles: $\theta_{1},\theta_{2},\ldots \theta_{k+1}$. These angles do not have to be uniformly distributed over 180°. They can, for example, be densely distributed in a very small angular range (say, 40°).

Thus, if an imaging system is able to measure all information of the object in the Fourier domain at (0, 0), including higher-order mixed partial derivatives at (0, 0), a Maclaurin series expansion can be formed; this expansion converges everywhere in the complex plane. Since this expansion converges, a truncated expansion (with finite number of terms) can be used to approximate the Fourier-domain function F(u,v).

The next step is to evaluate the truncated Maclaurin expansion of F(u,v) at any location (u,v). For example, we can evaluate the expansion at a regular grid of (u,v). Finally, we perform the 2D inverse Fourier transform to obtain the reconstructed image f(x,y).

The “entire function” idea presented above does not apply in the spatial domain. The spatial domain image cannot be an “entire function” because the spatial domain image has a finite support and thus the image must have discontinuities. Discontinuity prevents the spatial domain images from being differentiable everywhere.

In order to gain some intuitive about the feasibility whether a truncated Taylor series expansion is useful in image reconstruction, the next section will present a computer simulation example using an imperfect computer, which has limitations such as a finite-word length and round-off errors.

A square phantom f(x,y) has a known, close form 2D Fourier transform F(u,v), which is a product of two sinc functions:

(18) $$F(u,v)=\text{sinc}(a\cdot u)\cdot \text{sinc}(a\cdot v)\;=\frac{\text{sin}(a\cdot u)}{a\cdot u}\cdot \frac{\text{sin}(a\cdot v)}{a\cdot v},$$

where the parameter a determines the width of the square. It is known that the Maclaurin series expansion for the sine function

(19) $$sin(x)=\sum_{k=0}^{\infty }\frac{(-1{)}^{k}}{(2k+1)!}x^{2k+1}.$$

This immediately gives

(20) $$\text{sinc}(a\cdot u)=\frac{sin(a\cdot u)}{a\cdot u}=\sum_{k=0}^{\infty }\frac{(-1{)}^{k}}{(2k+1)!}(a\cdot u{)}^{2k}.$$

Therefore, (18) becomes

(21) $$F(u,v)=\sum_{n=0}^{\infty }\frac{(-1{)}^{n}(a\cdot u{)}^{2n}}{(2n+1)!}\sum_{m=0}^{\infty }\frac{(-1{)}^{m}(a\cdot v{)}^{2m}}{(2m+1)!}.$$

A truncated version of (21) is

(22) $$F(u,v)\approx \sum_{k=0}^{\infty }\sum_{\begin{array}{c} n+m=k\\ n\geq 0,m\geq 0 \end{array}}\frac{(-1{)}^{n+m}(a\cdot u{)}^{2n}(a\cdot v{)}^{2m}}{(2n+1)!(2m+1)!}.$$

## Results

**Figure 2:**
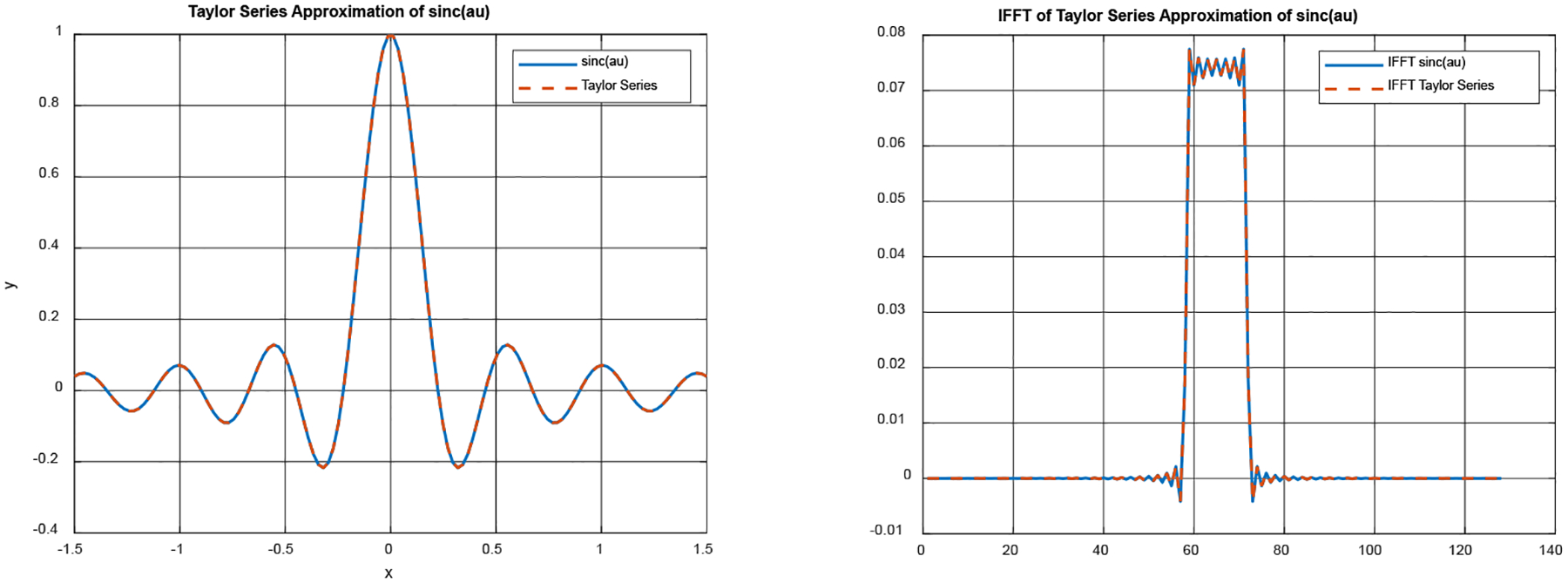
Left: 1D Fourier-domain signals (exact and approximate). Right: 1D IFFT versions for the two curves on the left.

A 1D Fourier transform pair is shown Figure 2, where the left diagram is in the 1D Fourier domain, and the right diagram is in the spatial domain. The left diagram contains two curves. The blue solid curve is a section of the sinc function. The orange broken curve is a truncated Maclaurin series expansion approximation with 50 terms of (20) and a=14. The right diagram also contains two curves. The blue solid curve is the 1D inverse Fourier transform of the section of the sinc function shown in the left diagram. The broken orange curve is the 1D inverse Fourier transform of the truncated Maclaurin series expansion shown in the left diagram. The 1D invers Fourier transform was implemented in MATLAB numerically using 128 discrete samples over [−1.5, 1.5] for the variable u. The MATLAB function ‘ifft’ was used in the computer simulation.

This 1D computer simulation demonstrates that the 50-term truncated Maclaurin series expansion is pretty close to the sinc function and that the discrete inverse Fourier transform can provide a pretty close approximation in the spatial domain. In other words, the “ifft” is not ill-conditioned.

**Figure 3:**
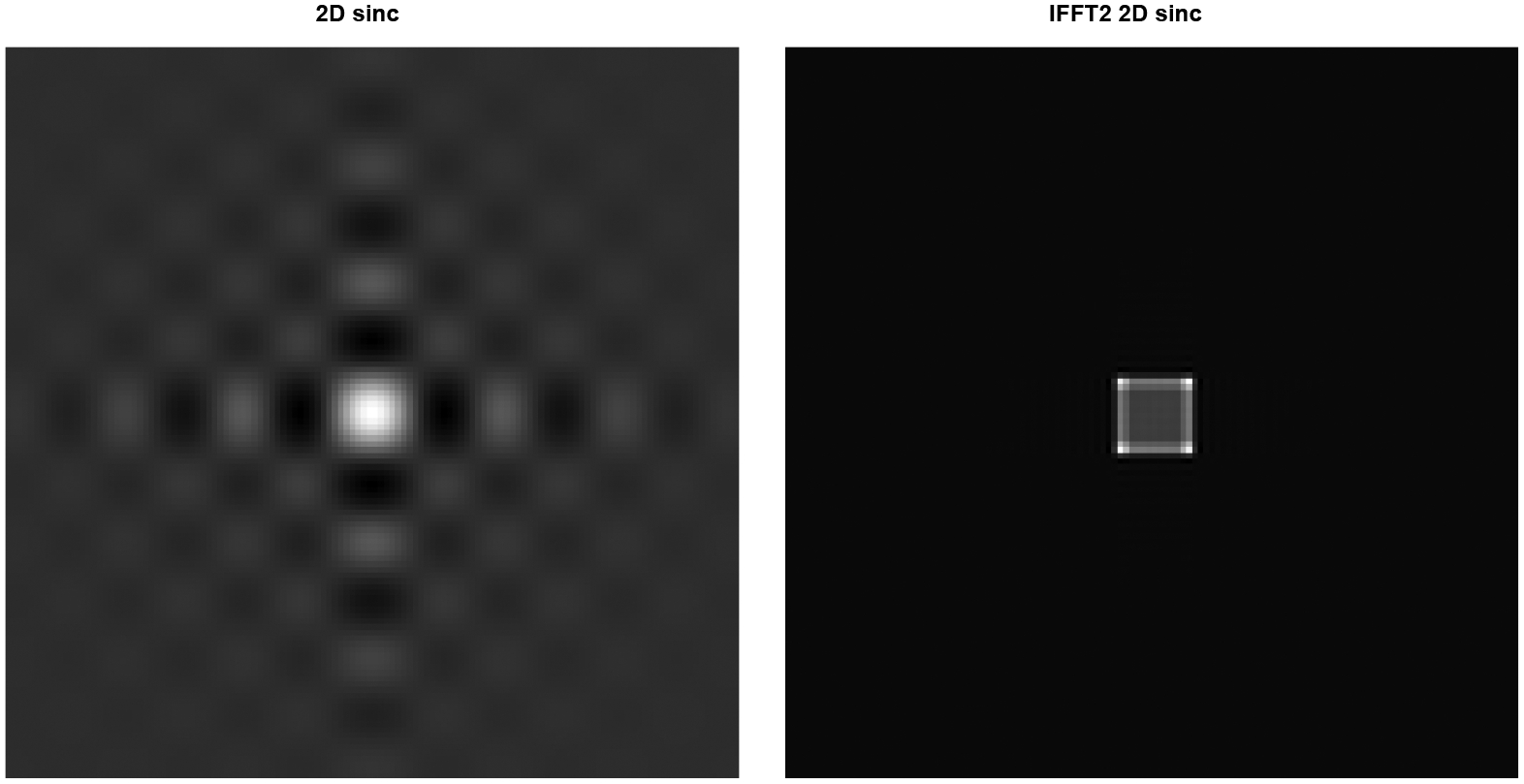
Left: 2D Fourier-domain image calculated by MATLAB’s built-in function. Right: 2D IFFT version for the image on the left.

A 2D Fourier transform pair is shown Figure 3, where the left diagram is F(u,v) in the 2D Fourier domain and the right diagram is its 2D inverse Fourier transform in the spatial domain. The object is the difference of two squares. The bigger square has a parameter a=14 and intensity of 1. The smaller square has a parameter a=10 and intensity of 0.7. Thus, F(u,v) is the difference of two 2D sinc functions and is calculated using MATLAB’s built-in sine function using discrete samples of u∈[-1.5,1.5] and v∈[-1.5,1.5], in a 128×128 regular grid. Taking MATLAB’s 2D inverse Fast Fourier Transform ‘ifft2,’ the left image F(u,v) becomes the right image f(x,y).

**Figure 4:**
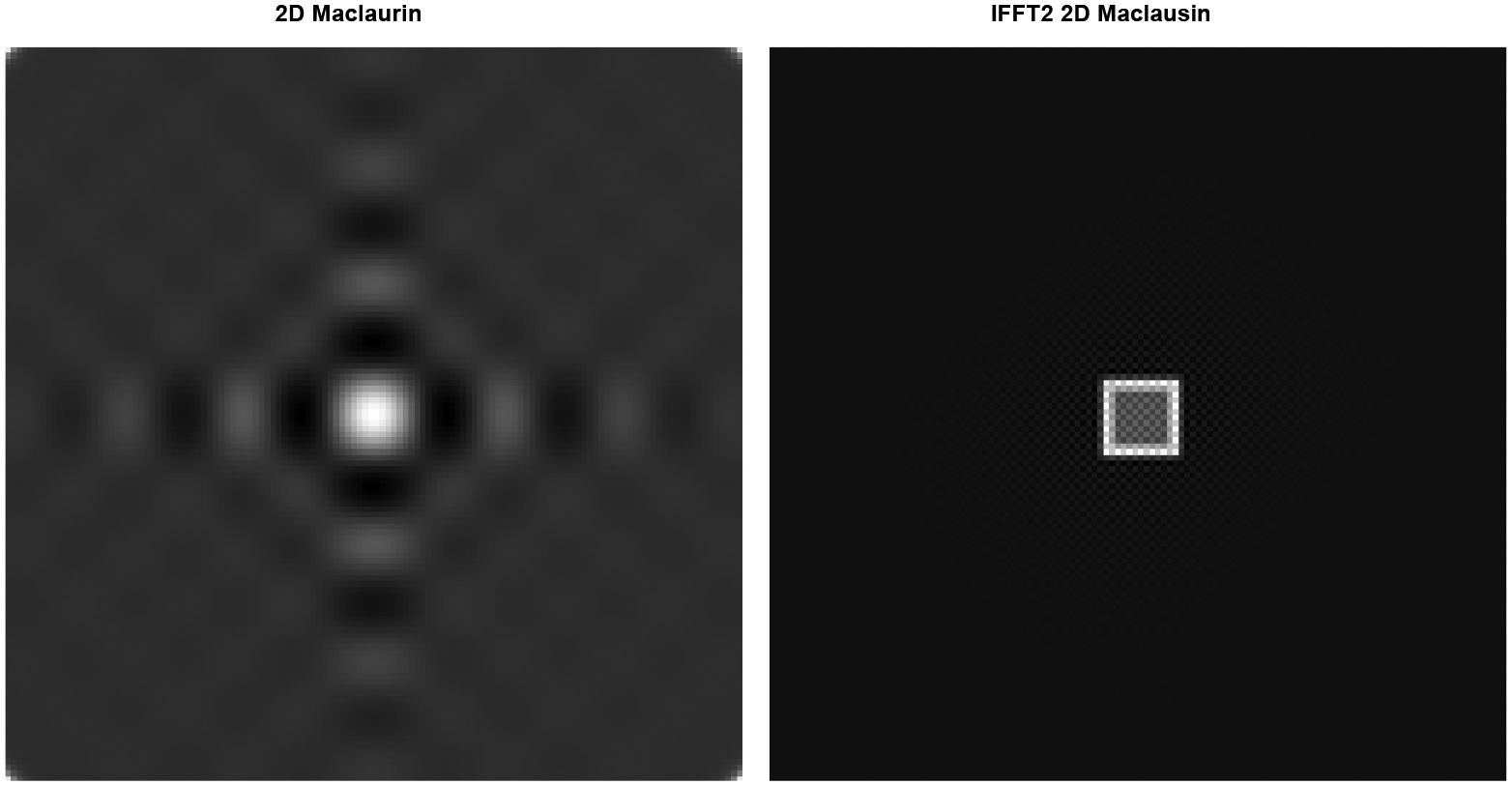
Left: 2D Fourier-domain image formed by a truncated 2D Maclaurin series expansion. Right: 2D IFFT version for the image on the left.

Figure 4 is almost the same as Figure 3, except that the Fourier domain image on the left is a truncated version of the Taylor series expansion (22) with a maximum value k being 50.

This 2D computer simulation demonstrates that the truncated Maclaurin series expansion is pretty close to the 2D sinc function and that the discrete 2D inverse Fourier transform can provide a pretty close approximation in the spatial domain. In other words, the “ifft2” is not ill-conditioned.

## Discussion and Conclusions

In the spatial domain, the patient image f(x,y) is a square-integrable function with a finite support. Its 2D Fourier transform F(u,v) is an entire function on the 2D complex plane. A Taylor series expansion at $\left(u_{0},v_{0}\right)$ converges everywhere in the complex Fourier space. The function F(u,v) can be evaluated with this Taylor series expansion anywhere in the complex Fourier space. The Taylor series expansion coefficients only depend on the local values of F(u,v) around $\left(u_{0},v_{0}\right)$. In other words, the spatial domain image f(x,y) can be reconstructed by the local information of F(u,v) in the Fourier domain. When $\left(u_{0},v_{0}\right)=(0,0)$, the expansion is referred to as the Maclaurin series expansion.

This paper assumes that the coefficients of the Taylor series expansion can be somehow exactly measured and calculated by a future hypothetical scanner. Then a truncated expansion can be formed and used for image reconstruction.

This paper considers image reconstruction only from the measurements of the object itself. Of course, prior information about the object can be useful to supplement unmeasured data. Machine learning is an excellent example of how prior information can make image reconstruction possible when measurements are incomplete. Some other prior information does not need neural network training, such as using the total variation (TV) norm minimization.

The robustness of the Taylor series expansion method is important but is beyond the scope of this paper. The singular value decomposition method has been used to study the ill-condition of limited angle tomography [62].

## Abbreviations

The following abbreviations are used in this manuscript:

1D: One dimensional
2D: Two dimensional
TV: Total variation
